# Extreme Lengthening of Tibia Using Bone Transport Technique: A Rare Case Report

**DOI:** 10.7759/cureus.32721

**Published:** 2022-12-20

**Authors:** Ankit M Jaiswal, Ratnakar Ambade, Aditya Pundkar, Pratik R Jaiswal, Madhu G Lakhwani

**Affiliations:** 1 Department of Orthopaedics, Jawaharlal Nehru Medical College, Datta Meghe Institute of Medical Sciences, Wardha, IND; 2 Department of Physiotherapy, Ravi Nair Physiotherapy College, Datta Meghe Institute of Medical Sciences, Wardha, IND; 3 Department of Musculoskeletal Physiotherapy, Ravi Nair Physiotherapy College, Datta Meghe Institute of Medical Sciences, Wardha, IND

**Keywords:** tibial fracture, external fixation, ilizarov technique, distraction osteogenesis, bone lengthening

## Abstract

This is a case describing a 13-year-old female student having a history of a fall-developed wound over the anterior aspect of the right leg with discharging sinus treated as chronic osteomyelitis and operated and distal tibia corticotomy and Ilizarov fixation was done. The patient was full of complications, but full limb lengthening was restored with follow-up. The Ilizarov frame's proper installation and the middle segment's efficient transportation are essential variables in reducing the likelihood of the transported segment deviating.

## Introduction

Treatment for severe limb bone abnormalities involves bone transfer. Ring or segmental external fixators used in conjunction with a solo or double corticotomy have a long history [1]. According to recorded examples, the largest tibial lengthening achieved with bifocal transportation was 22 cm, while the maximum lengthening achieved with unifocal transportation was 14.5 cm [2]. These two Ilizarov ring application does not offer the optimum structural setting for limb lengthening, as per Ilizarov's method [3]. The soft tissues of the posterolateral compartment create distraction-resisting pressures during tibial extension, resulting in valgus angulation. Although this occurs with the standard Ilizarov approach, the principle of distraction osteogenesis effectively maintains the alignment of the distracted bone [4]. A much more solid double-ring frame provides improved stability, leading to more favorable local conditions for bone regeneration than an unsteady two-ring lengthening framework, with further improvements in regeneration quality gained by minimizing damage to periosteal and endosteal blood supply at the site of the osteotomy [5].

## Case presentation

**Figure 1:**
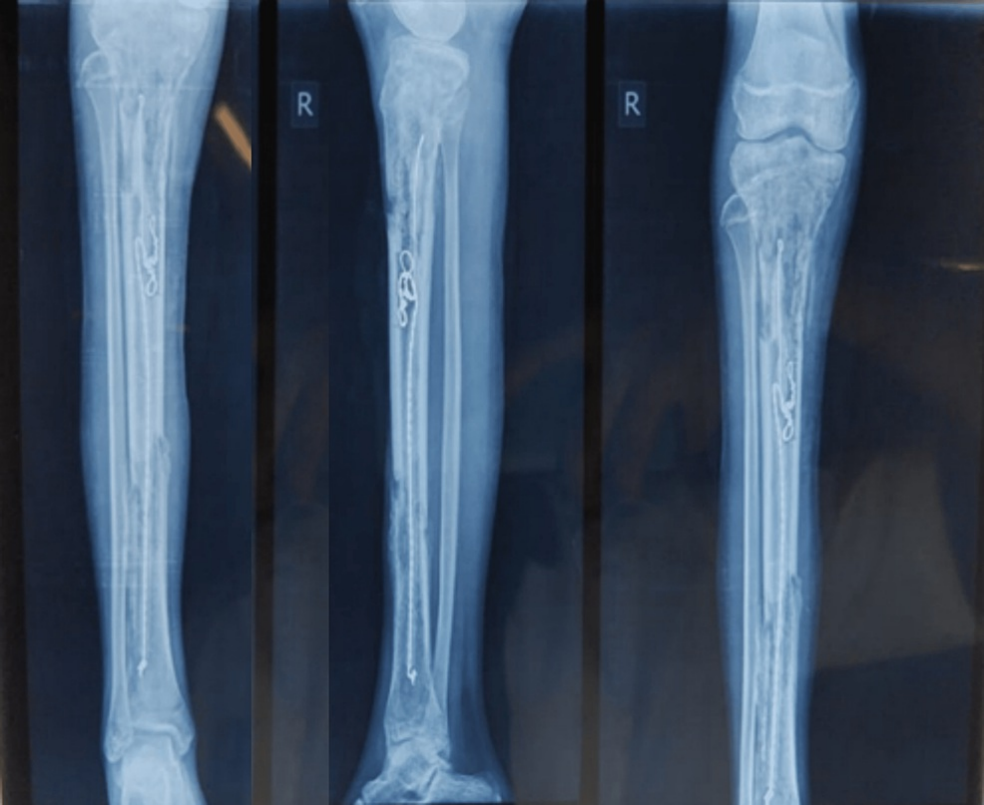
Cement-coated antibiotics beads were inserted in June 2020.

**Figure 2:**
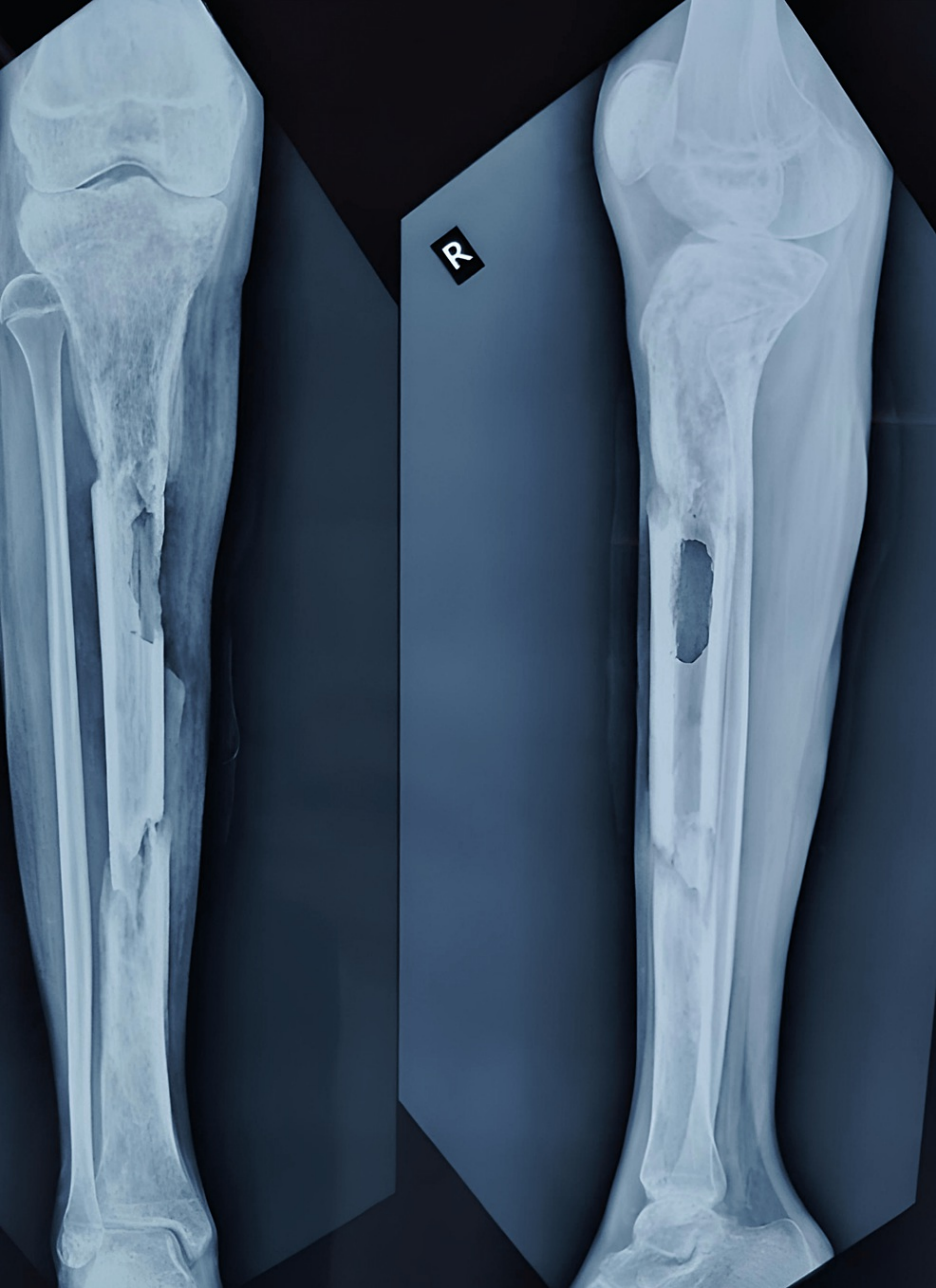
Antibiotics beads and stainless-steel wire were removed.

A 13-year-old female was brought to the outpatient orthopedic department with chief complaints of pain and swelling in her right leg in the last ten months. The patient gave an alleged history of slipping and falling on a wooden log while playing on her farm. The patient had direct blunt trauma to her right leg in May 2020. After falling, the patient had a sudden onset of pain followed by swelling that was progressive in nature dull aching, and localized to the right leg at the proximal portion. The patient also sustained a wound at the time of injury over the proximal third of the leg. Later, she developed pus at the wound site, i.e., the anterior aspect of the right leg, with discharging sinus. On June 3rd, 2020, she underwent incision and drainage and antibiotic bead application (Figure 1). Antibiotic bead removal was done on July 21, 2020 (Figure 2).

**Figure 3:**
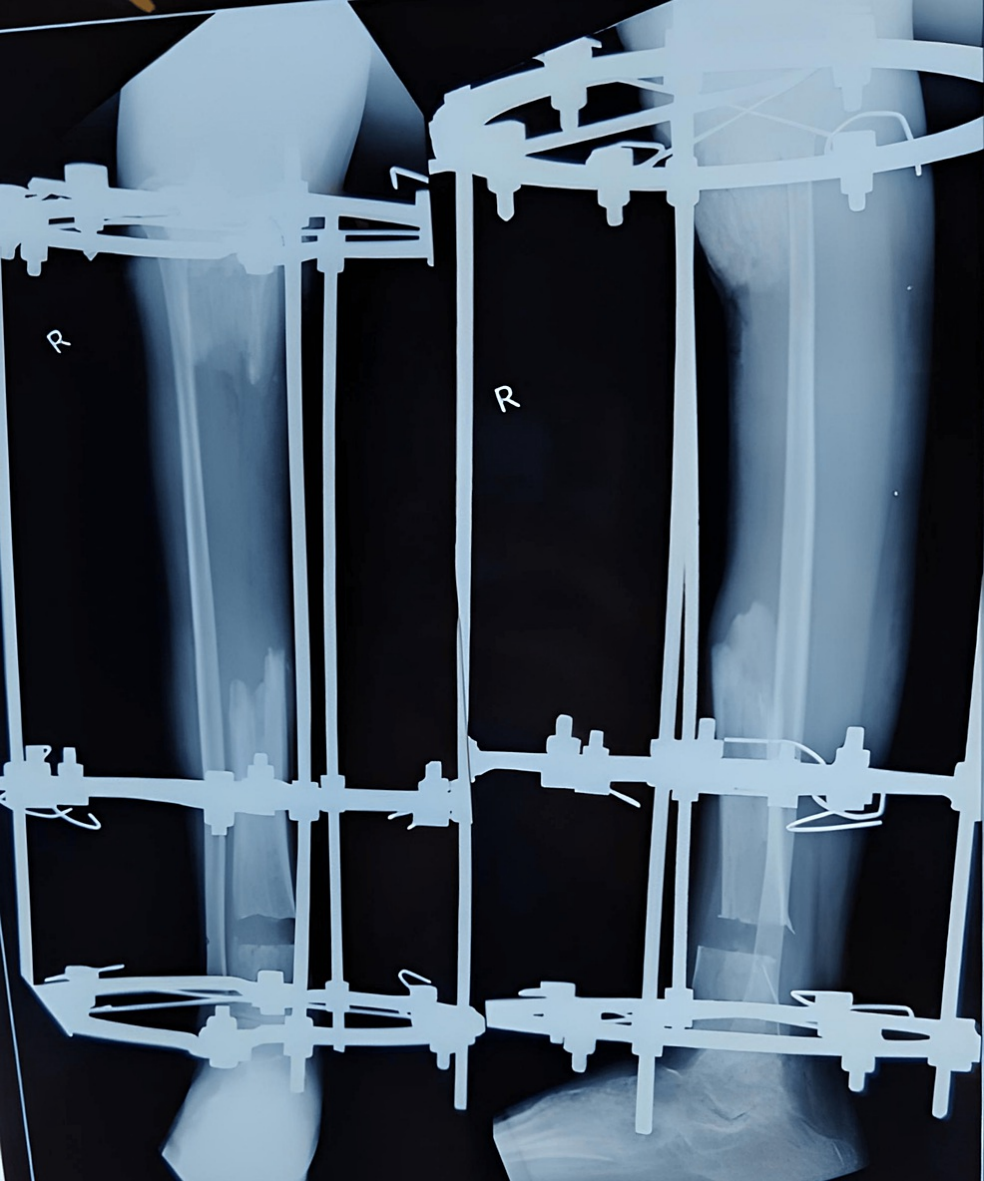
X-ray after corticotomy and Ilizarov application.

Further, the patient was diagnosed with chronic osteomyelitis and was operated on September 29, 2020, for the same. In this intervention, the sequestered bone was removed, and a distal tibial corticotomy and Ilizarov fixation was done. Three-ring fixation was done with six guide wires passing through bone, and the construct was connected with three AO Synthes connecting rods with two straight wires at each Ilizarov ring (Figure 3).

**Figure 4:**
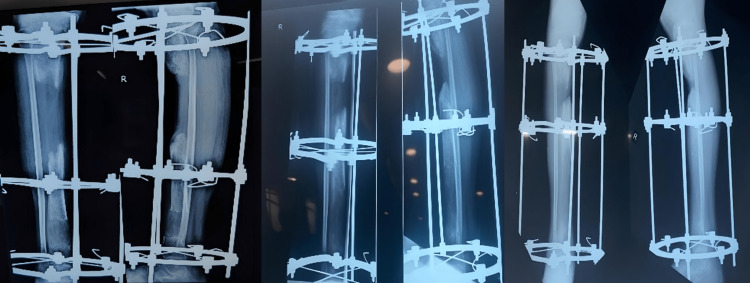
X-rays when the patient came for regular follow-ups subsequently every month.

Further distraction and compression were done on March 8, 2021; the skin that came in between bones was removed, and debridement of the fracture site was done. When the patient came for follow-up, realignment of the bony ends of the tibia was done and new rods were applied for better alignment (Figure 4).

**Figure 5:**
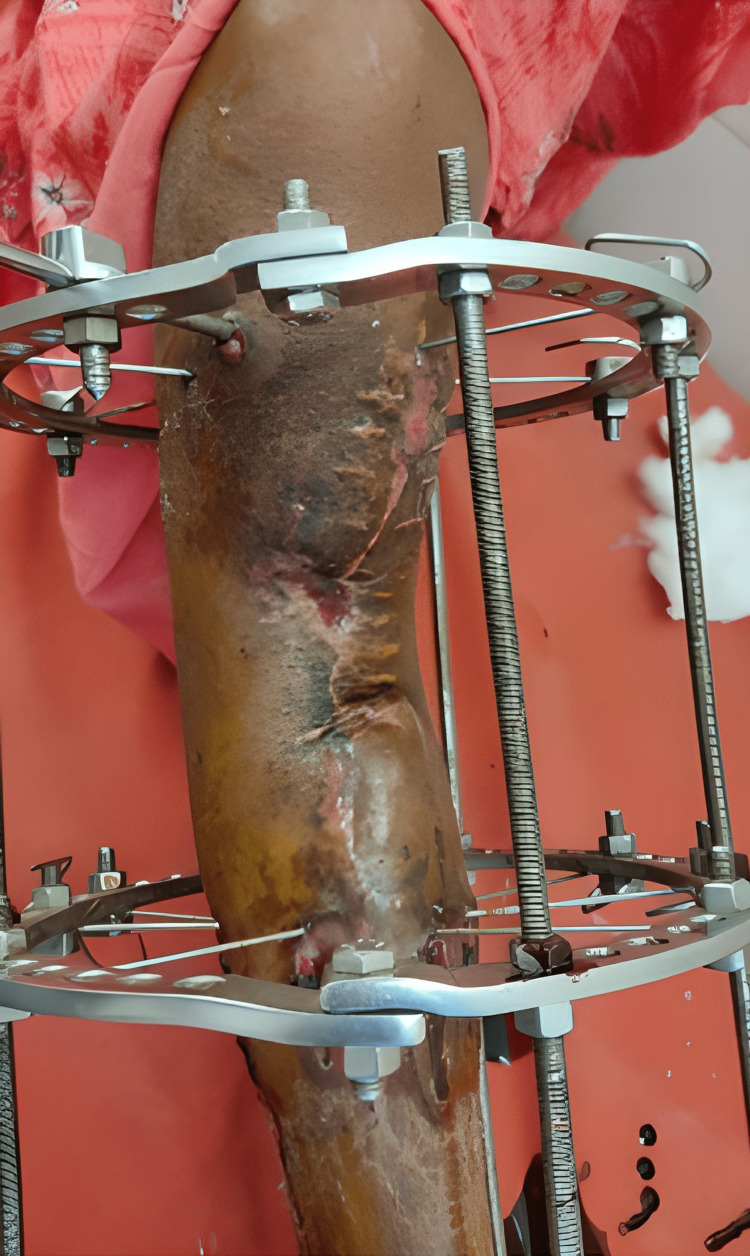
Clinical picture of when the patient comes for a follow-up in January 2021.

**Figure 6:**
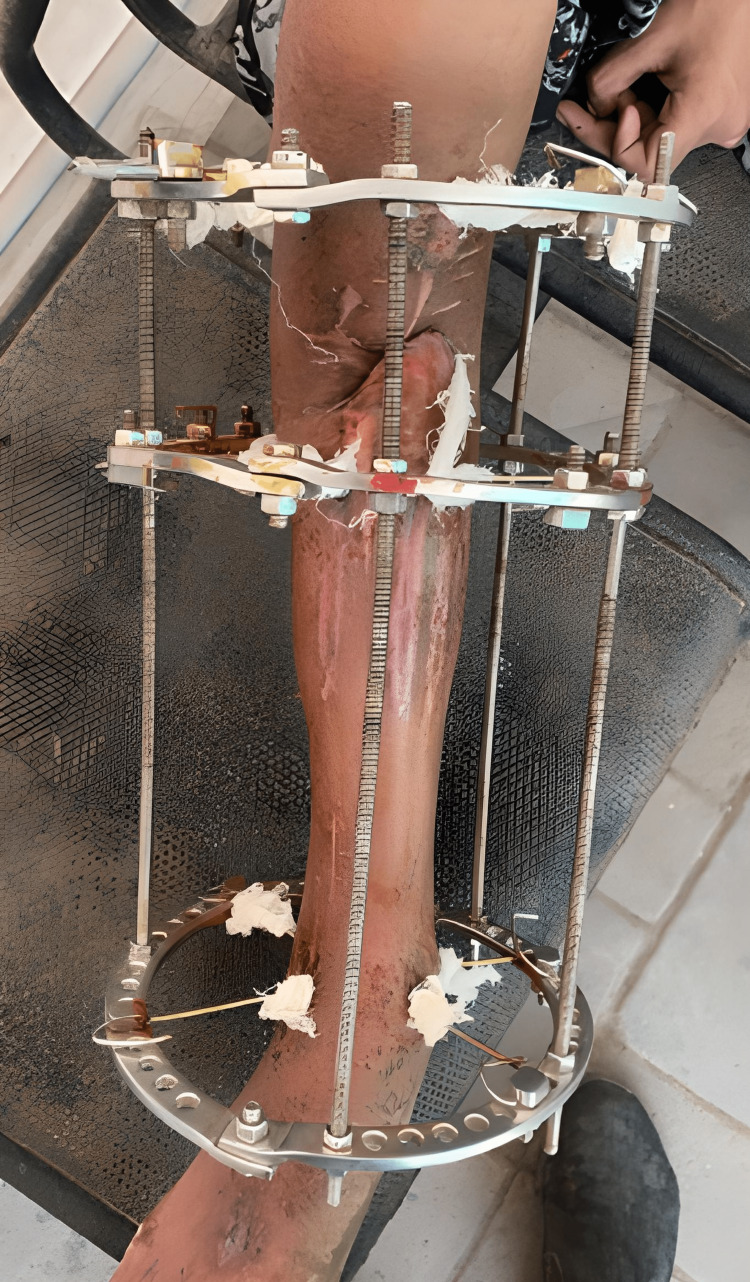
Clinical picture of when the patient came for a follow-up in March 2021.

**Figure 7:**
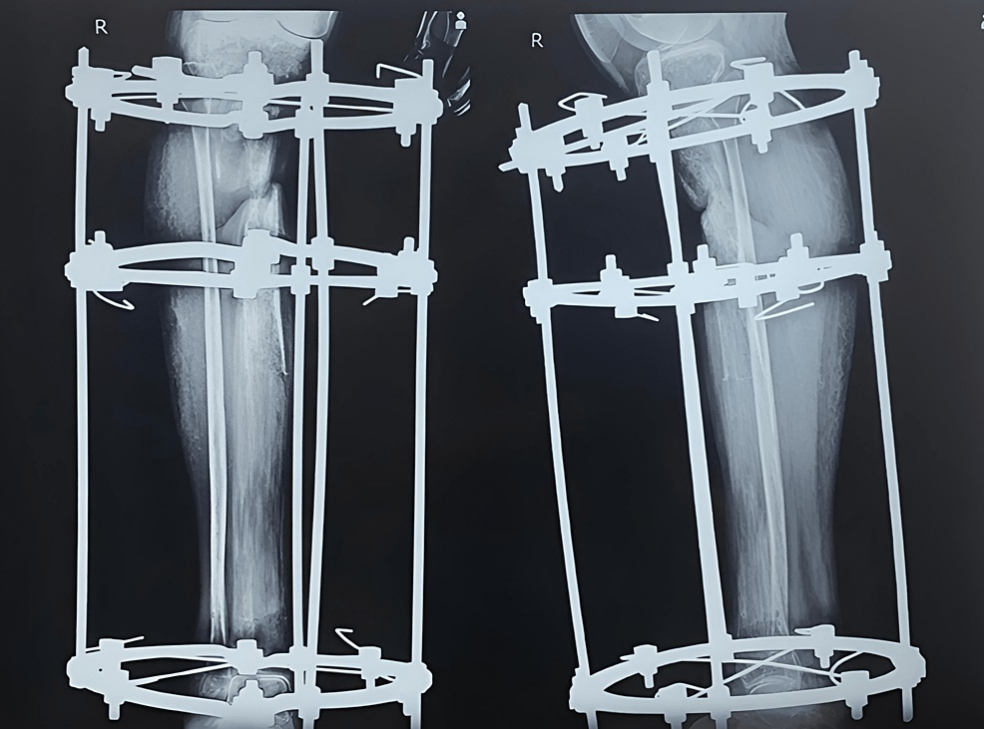
X-ray of follow-up on March 2021.

**Figure 8:**
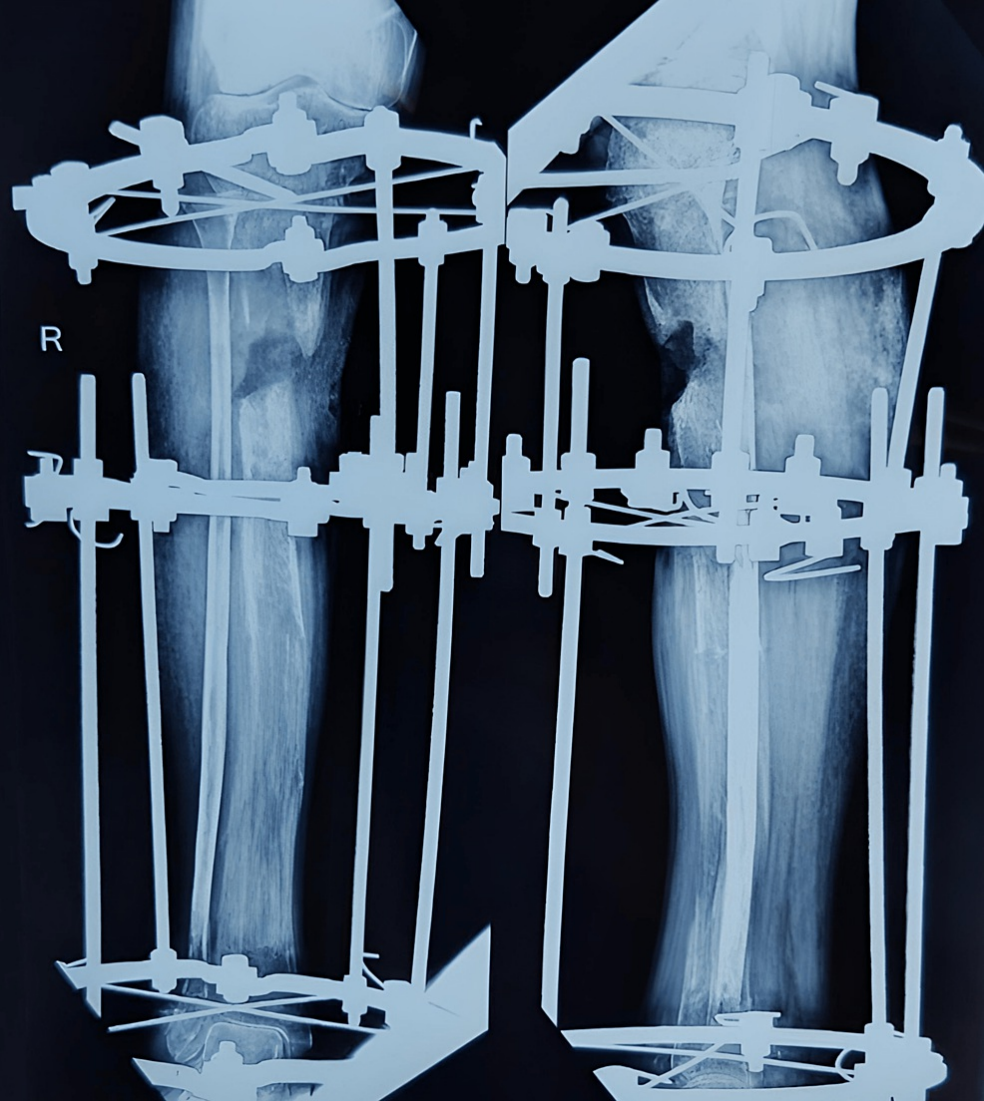
Realignment of external fixation was done.

**Figure 9:**
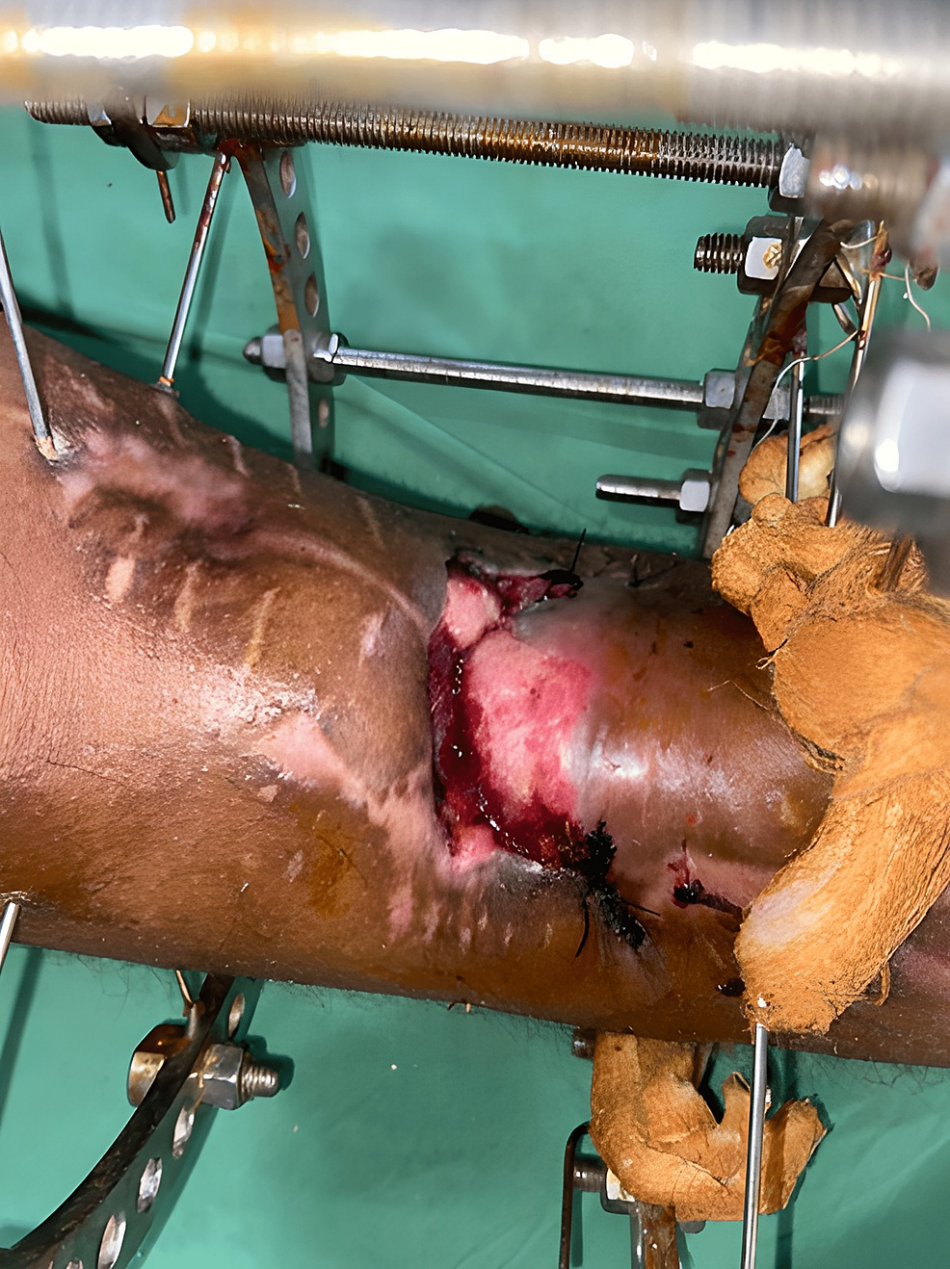
Postoperative clinical image of realignment and application of the Ilizarov fixator.

Following are the clinical images and x-rays (Figures 5, 6, 7) when the patient came for follow-up and the realignment of external fixators was done (Figures 8, 9).

We also received help from a physiotherapist, whose goals included patient's education, preserving cardiovascular fitness, enhancing strength, restoring joint range of motion, and gait training. Therapy began with isometric thigh workouts, training pelvic bridging, straight leg raises (SLR), and how to move or roll over in bed. Passive stretching of the muscles prevented the possibility of muscular shortening.

To preserve cardiovascular fitness, breathing exercises were given. Static activities for the quads and hams were also started, as well as SLR for the affected limb. For the patellar range of motion, patellar gliding was performed in the inferior, superior, and lateral directions. Stretches and ankle pumps were provided. When enough strength was gained sitting to standing was taught so that we can proceed to walk with the support of a walker. All this was done in a phase-wise manner by timely increasing the reps of the exercises.

Despite the fact that this case scenario was exceptionally difficult, the entire limb length was recovered.

## Discussion

The Ilizarov circular fixator is commonly used to treat tibial abnormalities. In the femur, monolateral fixators are preferred. It has been recommended that tibial defects longer than 6 cm should be treated by means of distraction techniques. Through tensile force or distraction tension stress, it aids in the regeneration of bone and tissue creation [6]. The external fixator creates a gap in the bone ends that will be filled with natural bone and tissue cells. This procedure necessitates time and periodic adjustments to the external fixator in order to promote progressive distraction and osteogenesis, as well as frequent corrections to the axis of the distracted bone. Based on the condition and the variety of Ilizarov approach employed, the authors saw higher numbers of therapeutic efficacy ranging from 77% to 100% [7]. Ilizarov procedures produced acceptable operational and bone results when used to treat critical-sized tibial bone defects (CSBDs) that were infectious or not. The most important step in treating bone infections is radical debridement [8].

## Conclusions

A reliable, less invasive method for the treatment of severe bone defects is the bone transportation procedure with Ilizarov exterior rings. In order to lower the possibility of the transferred segment diverging, the Ilizarov frame must be applied correctly and the center section must be carried well and continuous monitoring throughout the procedure is mandatory as well. Nevertheless, in an attempt to achieve the finest results, rigorous commitment from the patient is required in the form of routine follow-ups and screening.
